# Acknowledgement to Reviewers 2022

**DOI:** 10.3389/ijph.2023.1605946

**Published:** 2023-03-20

**Authors:** IJPH Editorial Office

**Affiliations:** Swiss School of Public Health (SSPH+), Zürich, Switzerland

The Editors of the *International Journal of Public Health* would like to thank all Reviewers in 2022 for the time they have spent and the valuable contributions they have made to ensure the scientific quality of the journal.

Gboyega Abikoye, Nigeria

Rufin Kouassi Assare, Côte d'Ivoire

Karim Abu-Omar, Germany

Shervin Assari, United States

Laura Acosta, Argentina

Nathalie Auger, Canada

Adekunle Adedeji, South Africa

Abolfazl Avan, Iran

Joko Adianto, Indonesia

Sina Azadnajafabad, Iran

Wignyo Adiyoso, Indonesia

Noshaba Aziz, China

Siddharth Agarwal, India

Adriana Baban, Romania

Claudia Agnoli, Italy

Gaudenz Bachmann, Switzerland

Prince Agwu, Nigeria

Georgian Badicu, Romania

Benojir Ahammed, Bangladesh

Sanghyuk Bae, Republic of Korea

Meraj Ahmad, Nepal

Ruhai Bai, China

Hedyeh Ahmadi, California

Franca Barbic, Italy

Mohammed Ahmed, Ethiopia

Chiara Barchielli, Italy

Felicity Aiano, Switzerland

Lillian Barra, Canada

Jennifer Ailshire, United States

Armando Basagoitia, Chile

Sedef Akgungor, Turkey

Osnat Bashkin, Israel

Yingigba Akinyemi, Nigeria

Marieke Battjes-Fries, Netherlands

Josleen Al Barathie, Lebanon

Martina Behanova, Austria

Jesus Alejandro Aldana Lopez, Mexico

Maria Bekker-Nielsen Dunbar, Switzerland

Mohammad Ali, Australia

David Bennett, United states

Karim Al-Jashamy, Malaysia

James Bentham, United Kingdom

Yagoub Al-Kandari, Kuwait

André Berchtold, Switzerland

Francisco Alonso, Spain

Anke Berger, Switzerland

Anmar Al-Taie, Turkey

Sverre Bergh, Norway

Rene Amalberti, France

Fabrizio Bert, Italy

Ankit Anand, India

Emmanuel Bhaskar, India

Italo F. Angelillo, Italy

Stella Bialous, United States

Daniela Anker, Switzerland

Monica Bianchi, Switzerland

Benedict Armstrong, United Kingdom

Shankar Biradar, India

Deodato Assanelli, Italy

Marie Bismark, Australia

Bijit Biswas, India

Juan Chaparro, Colombia

Sanda Block, United States

André Chappatte, Switzerland

Wolfgang Bödeker, Germany

Lorena Charrier, Italy

Yvonne Bohr, Canada

Helen Chen, Singapore

Letza Bojorquez, Mexico

Tuo-Yu Chen, Taiwan

Sorana D. Bolboaca, Romania

Zhangling Chen, Netherlands

Xavier Bosch-Capblanch, Switzerland

Wirawan Chinviriyasit, Thailand

Laura Botta, Italy

Virginia Chiocchia, Switzerland

Brett Bowman, South Africa

Francesco Chirico, Italy

Javier Boyas, United States

Peter Choate, Canada

Lutgarda Bozzetto, Italy

Patricia Chocano-Bedoya, Switzerland

Alain Braillon, Switzerland

Dmytro Chumachenko, Ukraine

Julie Brandy, United States

Marco Ciccone, Italy

Nathalie Brender, Switzerland

Anthony Coetzer-Liversage, United States

Richard Brou Yapi, Côte d'Ivoire

Hélène Colineaux, France

Dario Bruzzese, Italy

Cameron Comrie, United States

Michał Brzeziński, Poland

Alessio Conti, Italy

Patrick Brzoska, Germany

Andrea Tenorio Correia da Silva, Brazil

John Bua, Uganda

Alessandra Costanza, Italy

Jens Bucksch, Germany

Gonzalo Cuadra-Malinarich, Chile

Diana C. Buitrago Garcia, Switzerland

Leticia Cuellar, United States

Celia Burgaz, Belgium

Francy Cuervo, Colombia

Arndt Büssing, Germany

Stéphane Cullati, Switzerland

Yasmin Butt, United States

Katarzyna Czabanowska, Netherlands

Olena Bychkovska, Switzerland

Katarzyna Czech, Poland

Luis Calmeiro, United Kingdom

Goodarz Danaei, United States

Mabel Carabali, Canada

Zuzana Dankulincová, Slovakia

Irene Carrillo, Spain

BreAnne Danzi, United States

Rodrigo Casanueva, Chile

Hank Dart, United States

German Casas, Colombia

Sourav Das, India

Gemma Castaño-Vinyals, Spain

Hassan S. Dashti, United States

Magdalena Celuch, Finland

Jennifer Davis, Canada

Giulia Cesaroni, Italy

Chuan De Foo, Singapore

Qingsong Chang, China

Frank de Vocht, United Kingdom

Daniel Demant, Australia

Felix Ettensperger, Germany

Cesar Demayo, Philippines

Ikenna C Eze, Switzerland

Michael J. Deml, Switzerland

Ugochukwu Anthony Eze, Nigeria

Jiangli Di, China

Marta Fadda, Switzerland

Augusto Di Castelnuovo, Italy

Niki Fairhall, Australia

Mariachiara Di Cesare, United Kingdom

Nafis Faizi, Switzerland

Giuseppe Di Martino, Italy

Qiping Fan, United States

Anteo Di Napoli, Italy

Magdalena Fandino, United States

Adriano Dias, Brazil

Ya Fang, China

Jesús Díaz-Campo, Spain

Sasan Faridi, Iran

Hassan H. Dib, Canada

Mirko Farina, Russia

Paul Dietze, Australia

Mehdi Fazlzadeh, Iran

Derya Dikmen, Turkey

Svitlana Fedorenko, Ukraine

Bosiljka Djikanovic, Serbia

You-Shan Feng, Germany

Alejandro Domínguez-Rodríguez, Spain

Romulo Fernandes, Brazil

Keren Dopelt, Israel

Michelle Fernandez, Brazil

Julia Dratva, Switzerland

Luiz Fernando Machado, Brazil

Patrik Drid, Serbia

Giovanfrancesco Ferrari, Switzerland

Nichola Driver, Switzerland

Günther Fink, Switzerland

Ming-Jie Duan, Netherland

Maddalena Fiordelli, Switzerland

Duong Duc, Vietnam

Gilbert Fokou, Côte d'Ivoire

Simon Ducarroz, France

Sharon Fonn, Sweden

Alexandrine Dupras, Switzerland

Javier Fonseca, Colombia

Doris Duran, Canada

Mario Fordellone, Italy

Dick Durevall, Switzerland

Carlotta Franchi, Italy

Angel Dzhambov, Bulgaria

Rochelle Frounfelker, Canada

Tamer Edirne, Turkey

Jonila Gabrani, Switzerland

John Eisman, Australia

Septyanto Galan Prakoso, Indonesia

Stephanie Ejegi-Memeh, United Kingdom

Myriam Galfo, Italy

Laurie Elit, Canada

Azmat Gani, Oman

Karmen Erjavec, Slovenia

Ibza García, Mexico

Clémence Esse, Côte d'Ivoire

Jonny Alejandro García Luna, Colombia

Emmanuel Esso, Côte d'Ivoire

Pablo García-Molina, Switzerland

Dóra Eszter Várnai, Hungary

Antonio Gasparrini, United Kingdom

Teresa Gavaruzzi, Italy

Wojciech Hanke, Poland

Martina Gažarová, Slovakia

Sarah Hanson, United States

Armin Gemperli, Switzerland

Haijing Hao, United States

Başaran Gençdoğan, Turkey

Anthony Hauser, Switzerland

Hailay Gesesew, Australia

Margaret Haworth-Brockman, Canada

Teshome Geta, Ethiopia

Li He, China

Vida Ghasemi, Iran

Yini He, Switzerland

Ehsan Gholami, United States

Linnea Hedman, Sweden

Santu Ghosh, India

Björg Helgadóttir, Sweden

Anwal Ghulam, Italy

Timea Helter, Austria

Emilio Gianicolo, Germany

Alexander Henke, United States

Antonio Golpe, Spain

Aquiles Henriquez, Ecuador

Noha Gomaa, Canada

Lize Hermans, Belgium

Andrea Gómez, Ecuador

Daniel Hernández Calle, Switzerland

Juan Gómez-Salgado, Spain

Barbara Hoffmann, Germany

Judite Gonçalves, United Kingdom

Hans Hogerzeil, Netherlands

Gilberto Gonzalez-Parra, United States

Enayatollah Homaie Rad, Iran

Vijayaprasad Gopichandran, India

Kai Hong, United States

Dipti Govil, India

Helen Hong Chen, Canada

Rodrigo Goycolea, Chile

Brady Alfred Hooley, Switzerland

Jakub Grabowski, Poland

Mariko Hosozawa, Japan

Pablo Gracia, Ireland

Jian Hou, China

Stefan Gruber, Germany

Lei Hou, China

Hengyu Gu, China

Wan-Hua Hsieh, Taiwan

Leo Gugerty, United States

Chunyan Hu, China

Negnorogo Guindo-Coulibaly, Côte d'Ivoire

Dian Hu, United States

Sanjeev Gupta, India

Cassandra Hua, United States

Erni Gustina, Indonesia

Wenyu Huang, China

Henrike Häbel, Sweden

Yu-Te Huang, China

Guy Haller, Switzerland

Jimi Huh, United States

Mats Hallgren, Sweden

Chia Hui Tan, Taiwan

Scott B. Halstead, United States

Tim Huijts, Netherlands

Bing Han, China

Rola Husni, Lebanon

Ziqiang Han, China

Ellen Idler, United States

Kristen Jafflin, Switzerland

Phung Lang, Switzerland

Martin Janičko, Slovakia

Esther Lau, Switzerland

Eric Janssen, France

Guy Launoy, France

Bec Jenkinson, Australia

Marco Lauriola, Italy

Ayan Jha, United States

Hyo Lee, Republic of Korea

Anne Jörns, Germany

Jaesub Lee, United States

Julius Juodakis, Sweden

Munseob Lee, United States

Meliha Jusufoska, Switzerland

Wen-Chung Lee, Taiwan

Zubair Kabir, Ireland

Gobopamang Letamo, Botswana

Mariko Kanamori, Japan

Diane Levin-Zamir, Israel

Werner Karrer, Switzerland

Rosamund F. Lewis, Switzerland

Sakari Karvonen, Finland

Xiaoyan Li, China

Lisa Kawatsu, Japan

Zeyu Li, China

Mory Keita, Switzerland

Marta Lima-Serrano, Spain

Bernhard Kerschberger, Switzerland

Stephen Lindow, Ireland

Faham Khamesipour, Iran

Julia Liou, United States

Bilal Khodr, United States

Qimin Liu, United States

Biljana Kilibarda, Serbia

Tianyin Liu, China

Kyuwoong Kim, Republic of Korea

Erand Llanaj, Germany

Jonathan Klein, United States

Nathan Lo, United States

Casey Klofstad, United States

Pablo Lopez, Italy

Sarah Koens, Germany

Maichou Lor, United States

Christopher Kofahl, Germany

Elsa Lorthe, Switzerland

Kamyar Kompani, Switzerland

Kevin Lu, United States

Jaroslava Kopcakova, Slovakia

Henry Lucas, United Kingdom

Michaela Kosticova, Slovakia

Daniel Ludecke, Germany

Levente Kriston, Germany

Destiny Lutero, Philippines

Esther Kuenzli, Switzerland

Shingai Machingaidze, Netherlands

Nino Kuenzli, Switzerland

Putul Mahanta, India

Anggi Kurniadi, Switzerland

Farnaz Mahdavian, Germany

Sharon Sam Mee Kwan, Malaysia

Veronica Mamondi, Spain

Giuseppe La Torre, Italy

Maria Manaca, Mozambique

Samanta Lalla-Edward, South Africa

Erma Manoncourt, France

Prabhat Lamichhane, Australia

Tsegahun Manyazewal, Ethiopia

William Marelich, United States

Daniel Mork, United States

Jurgita Markeviciute, Lithuania

Zachary Morris, United States

Simon Marmet, Switzerland

Jose Moya, Chile

Jimmy Martin-Delgado, Ecuador

Rebeca Mozun, Switzerland

José Martínez, Switzerland

Holger Muehlan, Germany

María Paz Martínez Rubio, Chile

Paddington Tinashe Mundagowa, Zimbabue

Gonzalo Martinez-Ales, United States

Ainoa Muñoz San José, Spain

Daniela Martini, Italy

Karsten Münstedt, Switzerland

Maria Luisa Marván, Mexico

Joanna Myszkowska-Ryciak, Poland

Yousri Marzouki, Qatar

Tomio Nakayama, Japan

Rainier Masa, United States

Rebecca Nantanda, Uganda

Franco Mascayano, United States

Tiziana Nazio, Italy

Guido Mascialino, Ecuador

Antonio Neri, United States

Maria Masulli, Italy

Fiona Newton, Australia

Anna Vittoria Mattioli, Italy

Kandala Ngianga-Bakwin, United Kingdom

Fatemeh Mayvaneh, Iran

Nhung Nguyen, Vietnam

Joanna Mazur, Poland

Hannakaisa Niela-Vilen, Finland

Mary McENiry, United States

Dimitrios Nikolaou, United States

Lynn McIntyre, Canada

Yanping Niu, China

David McQueen, Switzerland

Darrell Norman Burrel, United States

Roberto Mediavilla, Spain

Rommy Helena Novoa Reyes, Peru

Esther María Medrano Sánchez, Spain

Ruxandra Oana Ciobanu, Switzerland

Guy Mélard, Belgium

Alexander Odaibo, Nigeria

Nora Mélard, Belgium

Jihyun Oh, Republic of Korea

Sonja Memedovic, United Kingdom

Adesola Ojo Ojoawo, Switzerland

Gwenn Menvielle, France

Harriet Okronipa, United States

Philip Merrigan, Canda

Mark Okwir, Uganda

Sonja Merten, Switzerland

Michael Adu Okyere, China

Ourohiré Millogo, Burkina Faso

Eduardo Oliveira, Brazil

Gretta Mohan, Switzerland

Vânia Oliveira, Brazil

Antonio Jesús Molina Fernández, Spain

Jennifer O'Loughlin, Canada

Jette Möller, Sweden

Elijah Onsomu, United States

Lucia Monacis, Italy

Kwaku Oppong Asante, Ghana

Jennifer Morales-Cruz, Puerto Rico

Stjepan Orešković, Croatia

Michel Oris, Switzerland

Tahereh Rahimi, Iran

Secil Ozkan, Turkey

Ossi Rahkonen, Finland

Raffaele Palladino, Italy

Md. Estiar Rahman, Bangladesh

Alejandra Paniagua-Avila, United States

Bako Rajaonah, France

Salvatore Panico, Italy

Marco Ramirez, Mexico

Jerico Pardosi, Australia

Otavio Ranzani, Spain

Etienne Pardoux, France

Karen Rascati, United States

Fabrizio Pasanisi, Italy

Jacques Raubenheimer, Australia

Ke Peng, China

Sofia Ravara, Portugal

Adjani Peralta, United States

David Reid, Switzerland

Julian Perelman, Portugal

Simon Rella, Austria

Jose Pereyra Rodriguez, Spain

Françoise Renard, Belgium

Giovanni Pes, Italy

Lise Renaud, Canada

Richard Peter, Germany

Thomas Resch, Austria

Dusan Petrovic, Switzerland

Mohammad Reza Rouhollahi, Iran

Cuong Pham, Vietnam

Joffre Rezende Filho, Brazil

Elena Philippou, Cyprus

Ana Ribeiro, Brazil

Massimo Pieri, Italy

Fulvio Ricceri, Italy

Giacomo Pignataro, Italy

Heiner Rindermann, Germany

Carla Pires, Portugal

Pierre-Yves Robillard, Réunion

Cristina Bianca Pocol, Romania

Jose A. Rodas, Ecuador

Ghislain Poda, Burkina Faso

Dominik Röding, Germany

Dan Poenaru, Canada

Karen Roos, United States

Ramesh Poluru, India

Aldo Rosano, Italy

Simon Porcher, France

Lisa Rosen, United States

George Pounis, Greece

Jaroslav Rosenberger, Slovakia

Clara Prada Sanabria, Brazil

Valentina Rotondi, Switzerland

Nurka Pranjic, Bosnia and Herzegovina

Ana Lídia Rouxinol-Dias, Portugal

Krzysztof Przewozniak, Poland

Nicole Rübsamen, Germany

Xue Qiuhong, China

Pier Luigi Sacco, Italy

Alessandro Quaglieri, Italy

Tani Sagna, Burkina Faso

Vlad Radoias, United States

Yuzuru Sakamoto, Japan

Ricardo Rafael, Brazil

Muhammad Salar Khan, United States

Iman Rahimi, Switzerland

Nurul Izzah Abdul Samad, Malaysia

Samitha Samanmalee Gowinnage, Sri Lanka

Steven Stack, United States

Hanen Samouda, Luxembourg

Dagmar Starke, Switzerland

Yana Samuel, United States

Malcolm Steinberg, Canada

Jamile Sanches Codogno, Brazil

Janina Steinert, Germany

Ahmed Sarki, Uganda

Peter Steinmann, Switzerland

Eleni Sazakli, Greece

Steven D. Stellman, United States

Diana Schow, United States

Christiane Stock, Germany

Jamie Seabrook, Canada

Dave Stynen, Netherlands

Dominika Seblova, United States

L. Suzanne Suggs, Switzerland

Ilana Seff, United States

Dewi Susanna, Indonesia

Kevin Selby, Switzerland

Ezra Susser, United States

Kanchana Sethanan, Switzerland

Mikael Svensson, United States

Maninder Setia, India

Zachary Swick, United States

Barbara Settles, United States

Giovanna Tagliabue, Italy

Jamal Shams, Iran

Paula Tallman, United States

Mansour Shamsipour, Iran

Yi-Roe Tan, Singapore

Ruth Sharabany, Switzerland

Junko Tanuma, Japan

Akira Shibanuma, Japan

Thamara Tapia-Munoz, United Kingdom

Su Shih-Yung, Taiwan

Hossain Taufiq, United States

Saber Shiripour, Iran

Batool Tayefi, Iran

Tigist Tadesse Shonte, Ethiopia

Mauricio Tec, United States

Vittorio Simeon, Italy

Fabrizio Tediosi, Switzerland

Aditya Singh, India

Jean Tenena Coulibaly, Côte d'Ivoire

Titiksha Sirari, India

Gudina Terefe, Ethiopia

Rico Januar Sitorus, Indonesia

Duncan C. Thomas, United States

Ivana Skoumalova, Slovakia

Yves Nathan Tah Tian Bi, Côte d'Ivoire

Nathan Smith, United States

Monica Ticlla, Switzerland

Shinichi Sobue, Japan

Ahmet Topuzoğlu, Turkey

María Soledad Burrone, Chile

Marta Torrijos, Spain

Nicolas Sommet, Switzerland

Dimiter Toshkov, Belgium

David Speed, Canada

Maria Triassi, Italy

Stefanie Sperlich, Germany

Sarvodaya Tripathy, India

Niko Speybroeck, United Kingdom

Lucetta Tsai, Taiwan

Αreti Spyropoulou, Greece

Yi-Lin Tsai, United States

Johan Van Der Heyden, Belgium

Joshua Watson, United States

Antje Van Der Zee-Neuen, Austria

Yaguang Wei, United States

Raf Van Gestel, Netherlands

Robert Wellman, United States

Edwin Van Leeuwen, United Kingdom

Ming Wen, United States

Samantha Vanderslott, United Kingdom

Dean Whitehead, Australia

Abraham Varghese, Oman

Restuning Widiasih, Indonesia

Marco Ventura, Italy

Lisa Susan Wieland, United States

Giovanni Veronesi, Italy

Klaus Wingenfeld, Germany

Stefano Verzillo, Italy

Changfa Xia, China

Carolina Vila-Chã, Portugal

Weia XIA, China

Pablo Villalobos Dintrans, Chile

Huang Xiaoqiong, China

Sebastian Villarroel, Chile

Luyao Xie, China

Aleksandar Višnjić, Serbia

Sophie Xuefei Wang, China

Rachel Vogel, United States

Neşe Yakşi, Turkey

Marcus Vollmer, Germany

Lawrence Yang, United States

Olaf von dem Knesebeck, Germany

Tianan Yang, China

Ondine Von Ehrenstein, United States

Yung-Chieh Yen, Taiwan

Peter Von Philipsborn, Germany

Jie Yin, China

Tido Von Schoen-Angerer, Switzerland

Ceren Ertan Yoruk, United States

Viktor von Wyl, Switzerland

Megan Young, United States

Nico Vonneilich, Germany

Ju Young Jung, Republic of Korea

Udaya Wagle, United States

Zhiyong Yu, China

Thet Wai New, Myanmar

Ivan Žežula, Slovakia

Peter Waiswa, Uganda

Thomas Hongjie Zhang, Malaysia

Bingyuan Wang, China

Jiawei Zhang, Denmark

Ge Wang, China

Lei Zhang, United States

Peizhong Peter Wang, Canada

Qian Zhang, United Kingdom

Shengnan Wang, China

Ruohao Zhang, United States

Xiaoming Wang, China

Stephen X. Zhang, Australia

Xin Wang, United States

Zhuoni Zhang, China

Xuemei Wang, China

Peiling Zhou, China

Zicheng Wang, China

Zhonggao Zhou, China

Philippe Wanner, Switzerland

Fangfang Zhu, Switzerland

Sharon Warren, Canada

Md. Ziaul Isam, Bangladesh

Hannah Ziobrowski, Switzerland

Kamila Zvolska, Czechia

